# LncRNA TP73-AS1 promotes oxidized low-density lipoprotein-induced apoptosis of endothelial cells in atherosclerosis by targeting the miR-654-3p/AKT3 axis

**DOI:** 10.1186/s11658-021-00264-x

**Published:** 2021-06-08

**Authors:** Jia Ni, Zhen Huang, Dan Wang

**Affiliations:** 1 Stomatological Hospital, Southern Medical University, No. 366 Jiangnan Avenue South, Haizhu District, Guangzhou City, Guangdong Province People’s Republic of China; 2 Peking University School of Stomatology, Beijing, 100081 People’s Republic of China

**Keywords:** TP73-AS1, miR-654-3p, AKT3, Atherosclerosis, Apoptosis

## Abstract

**Background:**

Although lncRNA TP73-AS1 has been shown to play important roles in various human diseases, its function in atherosclerosis (AS) remains unclear.

**Methods:**

Human aortic endothelial cells (HAECs) were treated with 50 μg/ml oxidized low-density lipoprotein (ox-LDL) to establish an atherosclerotic cell model. The expression of TP73-AS1, miR-654-3p and AKT3 was detected by qRT-PCR. Cell functions were evaluated CCK-8 assay and flow cytometry. The protein levels of apoptosis-related proteins were evaluated by western blot. The binding relationship among TP73-AS1, miR-654-3p and AKT3 was determined by bioinformatics analysis and luciferase reporter assay.

**Results:**

TP73-AS1 was upregulated and miR-654-3p was downregulated in ox-LDL treated HAECs. TP73-AS1 silencing and miR-654-3p mimics decreased the viability and inhibited apoptosis of ox-LDL treated HAECs, decreased the expression levels of c-caspase-9, c-caspase-3 and Bax, and increased Bcl-2 expression. In addition, miR-654-3p inhibitor significantly reversed the inhibitory effects of si-TP73-AS1 on cell viability and apoptosis. TP73-AS1 could positively regulate AKT3 through directly sponging miR-654-3p.

**Conclusion:**

TP73-AS1 promoted apoptosis of ox-LDL stimulated endothelial cells by targeting the miR-654-3p/AKT3 axis, suggesting that TP73-AS1 might be a potential target for AS treatment.

## Background

Cardiovascular diseases (CVDs) have become common killers, causing more than 17.3 million deaths every year worldwide [1]. Atherosclerosis (AS), a CVD and inflammatory disease, is involved in endothelial dysfunction [2]. Previous studies have revealed that the aberrant proliferation and apoptosis of aortic endothelial cells are associated with AS development [3]. Hence, well understanding the specific mechanisms involved in human aortic endothelial cells’ (HAECs) growth and apoptosis may contribute to developing novel therapies to prevent AS development.

Long noncoding RNAs (lncRNAs) with a length of approximately 200 nucleotides play essential regulatory functions in AS progression [4]. For example, H19 expression is upregulated during AS development, and H19 inhibition effectively enhances proliferation and inhibits apoptosis of HAECs [5]. NEAT1 knockdown suppresses the proliferation and promotes apoptosis of HAECs [6]. TP73-AS1, a newly identified lncRNA involved in development of various tumors, is highly expressed in ovarian cancer cell lines, and TP73-AS1 downregulation inhibits proliferation, invasion, and migration of the ovarian cancer cell line SKOV3 in vitro [7]. In addition, TP73-AS1 is upregulated in several types of human cancer, and TP73-AS1 knockdown effectively attenuates the progression of ovarian cancer [7], hepatocellular carcinoma [8], and bladder cancer [9]. Based on the crucial roles of TP73-AS1 in human cancers, it might have a potential function in other diseases, such as AS. Thus, this study aimed to explore its potential functions in AS.

MicroRNAs (miRNAs) are small noncoding RNAs (ncRNAs) with a length of 22 nt, which regulate gene expression at the post-transcriptional level [10]. MiRNAs could control a series of AS pathological processes, including lipoprotein metabolism, endothelial cell growth and apoptosis, as well as immune responses [11]. For example, miR-654-3p suppresses tumor proliferation, migration, invasion, and epithelial-mesenchymal transition (EMT) in hepatocellular carcinoma [12], colon cancer [13], and gastric cancer [14]. MiR-654-3p was closely involved in the inflammatory responses through modulating RAB22A [15] in AS, indicating a potential role of miR-654-3p in AS.

AKT serine/threonine kinase 3 (AKT3) plays essential functions in many cellular processes, including cell growth, differentiation, proliferation, and apoptosis [16]. AKT3 promotes apoptosis of different types of tumor cells such as gastric cancer [17], endometrial carcinoma [18] and breast cancer [19]. AKT3 overexpression promotes the progression of esophageal squamous cell carcinoma and downregulates the expression level of apoptosis-related proteins including c-caspase-3, c-caspase-9, Bax and Bcl-2 [20]. AKT3 is highly expressed during AS progression and AKT3 kinase inhibits the pinocytosis of oxidized low-density lipoprotein (ox-LDL) in macrophages [21]. These studies indicate that targeting AKT3 might be a potential treatment strategy for preventing human diseases, including AS.

This study established an atherosclerotic cell model using 50 μg/ml ox-LDL treatment as previously reported [22–24] and found that TP73-AS1 was highly expressed in 50 μg/ml ox-LDL treated HAECs. In addition, TP73-AS1 knockdown and miR-654-3p overexpression effectively enhanced viability and inhibited apoptosis of ox-LDL treated HAECs. Furthermore, our results revealed a new regulatory network involved in AS development, i.e. silencing TP73-3P attenuated AS progression via inhibiting aortic endothelial cell apoptosis by targeting the miR-654-3p/AKT3 axis. We believe that our results provided a potential target of AS treatment.

## Materials and methods

### Cell culture

Human aortic endothelial cells (HAECs) were purchased from Platts Life Technology Co., Ltd. (Wuhan, China), and cultured in endothelial cell culture medium (Gibco, USA) supplemented with endothelial cell growth factor and 5% FBS at 37 °C with 5% CO_2_. When needed, 50 μg/ml ox-LDL was added for 24 h.

### Cell transfection

Small interfering RNA targeting TP73-AS1 (si-TP73-AS1, sense 5ʹ-CCCAGUGGUGACUCCACAATT-3ʹ and antisense: 5ʹ-UGGGUUAGGCCCCACCU GGTT-3ʹ) and corresponding negative control (si-NC, 5ʹ-ATTGGAAGCTGTGTTCCA TTA-3ʹ) were synthesized by Jima Biotech (Shanghai, China) and transfected into HAECs using Lipofectamine 2000 (Invitrogen, Carlsbad, California, U.S.A.). MiR-654-3p mimic, miR-NC, miR-654-3p inhibitor, inhibitor NC, TP73-AS1 overexpressing vector pc-TP73-AS1, AKT3 overexpressing vector pc-AKT3 and negative control vector pc-NC were purchased from RiboBio (Guangzhou, China) and transfected into HAECs using Lipofectamine 2000 following the manufacturer’s instructions.

### QRT-PCR

Total RNA was extracted using TRIzol reagent (Biosntech, Beijing, China). QRT-PCR analysis was performed on the ABI 7500 real-time PCR system using the SYBR Green PCR Kit (Takara Bio). U6 and GAPDH were regarded as internal references. The relative expression was calculated using the 2^−△△Ct^ method. The primers were TP73-AS1 forward 5ʹ-CCTTAATACCTGGGCCGGA-3ʹ and reverse: 5ʹ-CGGTA AGGAATCCCAAG-3ʹ; miR-654-3p forward 5ʹ-GGGCCAGTAATTGCACCTGTC-3ʹ and reverse 5ʹ-CCAGTGCAGGTTCCGAAGTA-3ʹ; AKT3 forward 5ʹ-GAACAAG GCTACTCGCCAAG-3ʹ and reverse 5ʹ-AAGGGCCAGTTGACGAAAC-3ʹ; GAPDH forward 5ʹ-TGGATTTGGACGCATTGGTC-3ʹ and reverse 5ʹ-TTTGCACTGGTAC GTGTTGAT-3ʹ; U6 forward 5ʹ-CTCGCTTCGGCAGCACA-3ʹ and reverse: 5ʹ-AACG CTTCACGAATTTGCGT-3ʹ.

### Western blot

Total protein was extracted using RIPA buffer. Approximately equal amounts of proteins were separated by 10% SDS-PAGE and transferred onto PVDF membranes (Bio-Rad). The membranes were incubated with primary antibodies against AKT3 (1: 1000, Lianmai, Shanghai, China), c-caspase-3 (1: 1000, Lianmai, Shanghai, China), c-caspase-9 (1: 1000, Lianmai, Shanghai, China), Bax (1: 1000, Lianmai, Shanghai, China), Bcl-2 (1: 1000, Lianmai, Shanghai, China) and GAPDH (1: 1000, Lianmai, Shanghai, China) overnight at 4 °C. Finally, the bands were visualized by an enhanced chemiluminescence (ECL) kit.

### CCK-8 assay

Briefly, 1 × 10^4^ HAECs were seeded into 96-well plates overnight. 10 µl of CCK-8 solution (Cell Counting Kit-8) was added to each well following transfection for 0, 24, 48, 72 and 96 h, and incubated for another 2 h. Finally, the absorbance was detected at 450 nm using a microplate reader.

### Apoptosis analysis

Cells apoptosis was examined by an Annexin V FITC/PI apoptosis detection kit purchased from Vazyme Biotech Co., Ltd. Briefly, HAECs were re-suspended in 1 × binding buffer (100 µl), and incubated with 5 µl of PI and 5 µl of Annexin V FITC in darkness for 10–20 min. The apoptotic rate was detected using flow cytometry (Jiyuan, Guangzhou, China).

### Luciferase activity assay

Luciferase reporter assay was performed as previously reported [25]. TP73-AS1 wild-type (WT) or mutant (MUT) or AKT3 containing the putative miR-654-3p binding site was synthesized and cloned into luciferase reporter pGL3 Basic vector (Promega). MiR-654-3p mimics or miR-NC was co-transfected with recombinant luciferase reporter plasmids into HAECs using Lipofectamine 2000. After 48 h for transfection, the relative luciferase activity was detected.

### RIP assay

RIP assay was performed using a commercial EZ-Magna RIP kit from EMD Millipore using Ago2 antibody (ab32381; 1:150; Abcam). In brief, HAECs were lysed and incubated with anti-IgG antibody (12-370; 1:150; Merck Millipore) or Ago2 antibody-conjugated protein A/G magnetic beads. Finally, RNAs were purified and analyzed by qRT-PCR.

### Statistical analysis

Data were presented as the mean ± standard deviation (SD). Differences were determined using Student’s *t*-test (between two groups) or one-way analysis of variance (ANOVA) (among multiple groups). *p* < 0.05 was considered as the significant threshold.

## Results

### TP73-AS1 was upregulated and miR-654-3p was downregulated in ox-LDL-treated endothelial cells

**Fig. 1:**
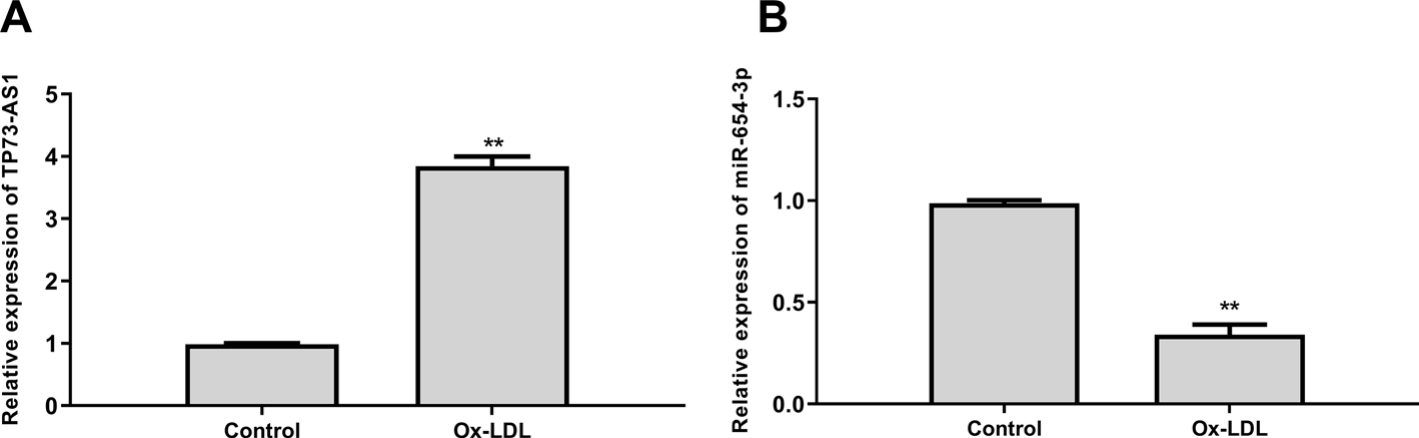
TP73-AS1 was upregulated and miR-654-3p was downregulated in ox-LDL-treated endothelial cells. The figure shows the levels of TP73-AS1 (**A**) and miR-654-3p (**B**) detected by qRT-PCR in HAECs treated with or without 50 μg/ml ox-LDL for 24 h. **P < 0.01

Firstly, HAECs were treated with 50 μg/ml ox-LDL for 24 h. QRT-PCR analysis showed that TP73-AS1 was significantly upregulated (*p* < 0.01) (Fig. 1A) and miR-654-3p was downregulated (*p* < 0.01, Fig. 1B) in ox-LDL-treated HAECs. These results suggested that TP73-AS1 and miR-654-3p might play important roles in AS.

### TP73-AS1 knockdown inhibited apoptosis of ox-LDL treated HAECs

**Fig. 2:**
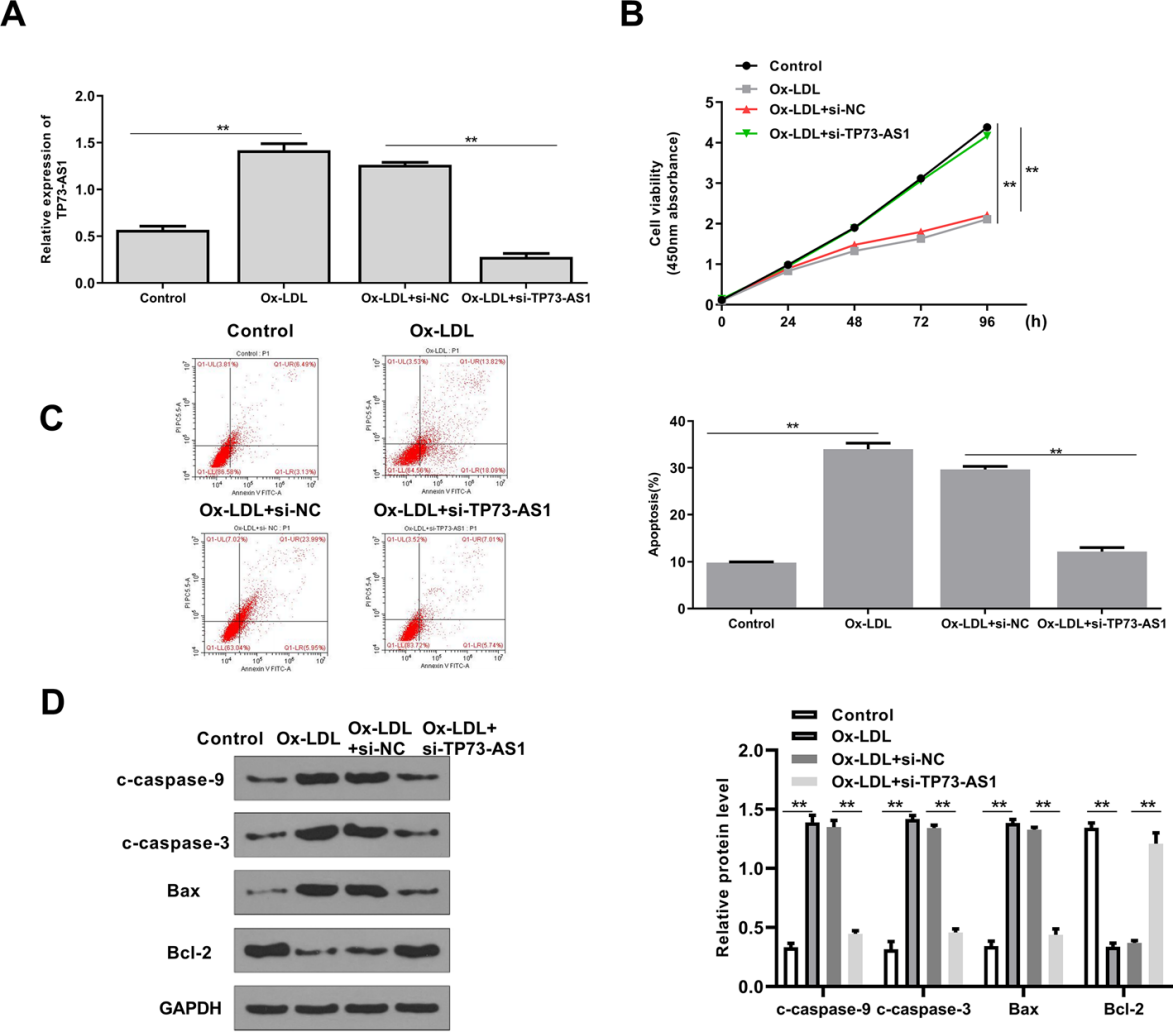
TP73-AS1 knockdown inhibited apoptosis of ox-LDL treated HAECs. The figure shows **A** TP73-AS1 levels evaluated by qRT-PCR, **B** cell viability assessed by CCK-8 assay, **C** apoptosis evaluated by flow cytometry and **D** the levels of apoptosis-related proteins detected by Western blot in HEACs transfected with si-TP73-AS1 or si-NC, and treated with 50 μg/ml ox-LDL for 24 h. **P < 0.01

To investigate TP73-AS1’s function, HAECs were transfected with si-TP73-AS1, and treated with ox-LDL. QRT-PCR analysis revealed that ox-LDL treatment significantly increased the TP73-AS1 level (*p* < 0.01), and si-TP73-AS1 markedly decreased the TP73-AS1 level in ox-LDL treated HEACs (*p* < 0.01) (Fig. 2A). CCK-8 assay indicated that ox-LDL treatment inhibited cell proliferation (*p* < 0.01), and si-TP73-AS1 significantly attenuated ox-LDL induced growth defection of HEACs (*p* < 0.01) (Fig. 2B). Ox-LDL treatment promoted apoptosis of HEACs (*p* < 0.01), and si-TP73-AS1 significantly inhibited ox-LDL-induced apoptosis of HEACs (*p* < 0.01) (Fig. 2C). Meanwhile, Western blot indicated that ox-LDL treatment significantly increased the protein levels of c-caspase-9, c-caspase-3, and Bax and decreased the Bal-2 level (all *p* < 0.01), while si-TP73-AS1 downregulated c-caspase-9, c-caspase-3, and Bax, and upregulated Bcl-2 (all *p* < 0.01) in ox-LDL treated HEACs (Fig. 2D). These data suggested that TP73-AS1 downregulation attenuated ox-LDL induced apoptosis in endothelial cells.

### Overexpression of miR-654-3p inhibited apoptosis of ox-LDL treated HAECs

**Fig. 3:**
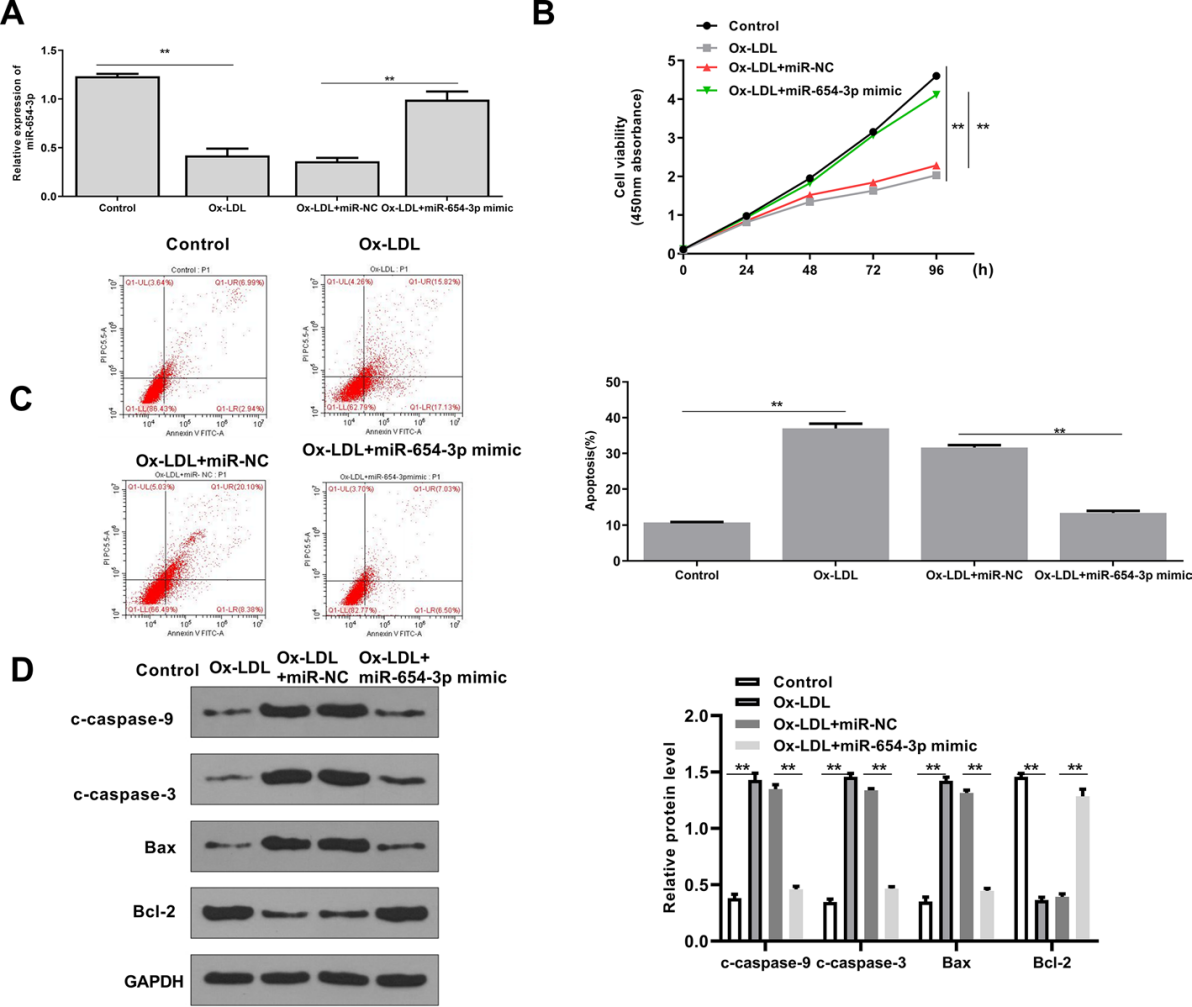
MiR-654-3p mimics inhibited apoptosis of ox-LDL treated HAECs. The figure shows **A** miR-654-3p level evaluated by qRT-PCR, **B** cell viability assessed by CCK-8 assay, **C** apoptosis evaluated by flow cytometry and **D** the levels of apoptosis-related proteins detected by Western blot in HEACs transfected with miR-654-3p mimics or miR-NC, and treated with 50 μg/ml ox-LDL for 24 h. **P < 0.01

To further explore the role of TP73-AS1 in AS, miR-654-3p overexpression was conducted in ox-LDL treated HAECs. Ox-LDL treatment decreased miR-654-3p level (*p* < 0.01), and miR-654-3p mimics significantly increased the miR-654-3p level in ox-LDL treated HEACs (*p* < 0.01) (Fig. 3A). Ox-LDL treatment inhibited cell proliferation (*p* < 0.01), and miR-654-3p mimics significantly inhibited ox-LDL induced growth defection of HEACs (*p* < 0.01) (Fig. 3B). Flow cytometry showed that ox-LDL treatment promoted apoptosis of HEACs (*p* < 0.01), and miR-654-3p mimics significantly inhibited ox-LDL induced apoptosis of HEACs (*p* < 0.01) (Fig. 3C). In addition, Western blot indicated that ox-LDL treatment significantly increased the protein levels of c-caspase-9, c-caspase-3, Bax, and decreased Bal-2 level (all *p* < 0.01), while miR-654-3p mimics downregulated c-caspase-9, c-caspase-3, Bax, and upregulated Bcl-2 (all *p* < 0.01) in ox-LDL treated HEACs (Fig. 3D). These results suggested that miR-654-3p mimics had a similar effect to si-TP73-AS1, and miR-654-3p overexpression inhibited apoptosis of ox-LDL treated HAECs.

### TP73-AS1 was a sponge of miR-654-3p

**Fig. 4:**
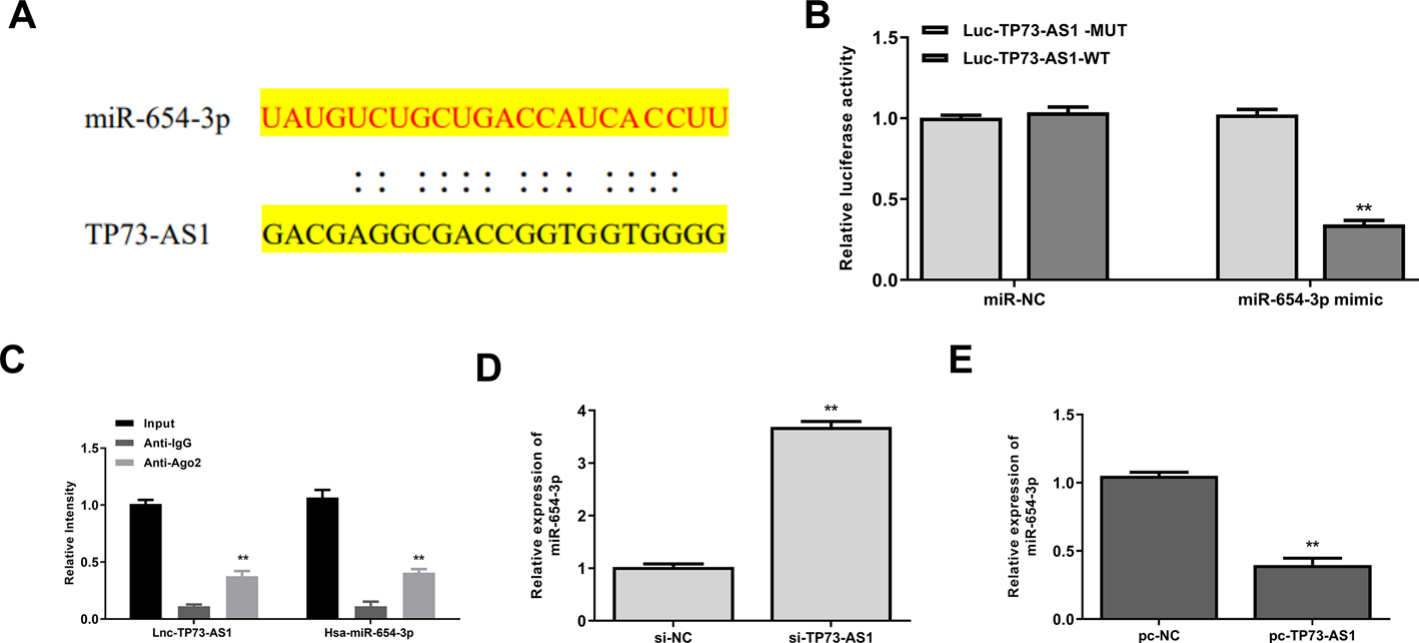
TP73-AS1 was a sponge of miR-654-3p. **A** The putative binding site between TP73-AS1 and miR-654-3p predicted by StarBase. **B** Results of dual luciferase reporter assay. **C** Enrichment of TP73-AS1 and miR-654-3p in miRNPs determined by RIP assay using anti-Ago2 antibody. **D** MiR-654-3p expression detected by qRT-PCR in HAECs transfected with si-TP73-AS1 or si-NC. **E** MiR-654-3p level detected by qRT-PCR in HAECs transfected with pc-TP73-AS1. **P < 0.01

To investigate the mechanism of TP73-AS1, the StarBase online database was used to predict the target miRNAs of TP73-AS1. The results indicated that miR-654-3p was a putative target of TP73-AS1 (Fig. 4A). Luciferase reporter assay showed that miR-654-3p mimics significantly decreased the relative luciferase activity of WT TP73-AS1 *(p* < 0.01), while having no effect on MUT TP73-AS1 (Fig. 4B). RIP assay demonstrated that TP73-AS1 and miR-654-3p were both enriched significantly in miRNPs containing Ago2 compared with those containing anti-IgG (*p* < 0.01) (Fig. 4C). Moreover, si-TP73-AS1 significantly increased the miR-654-3p level compared with si-NC in HAECs (*p* < 0.01) (Fig. 4D). TP73-AS1 overexpression (pc-TP73-AS1) markedly decreased miR-654-3p expression compared with pc-NC in HAECs (*p* < 0.01) (Fig. 4E). These results indicated that TP73-AS1 was a sponge of miR-654-3p.

### TP73-AS1 knockdown inhibited apoptosis of ox-LDL treated HAECs by targeting miR-654-3p

**Fig. 5:**
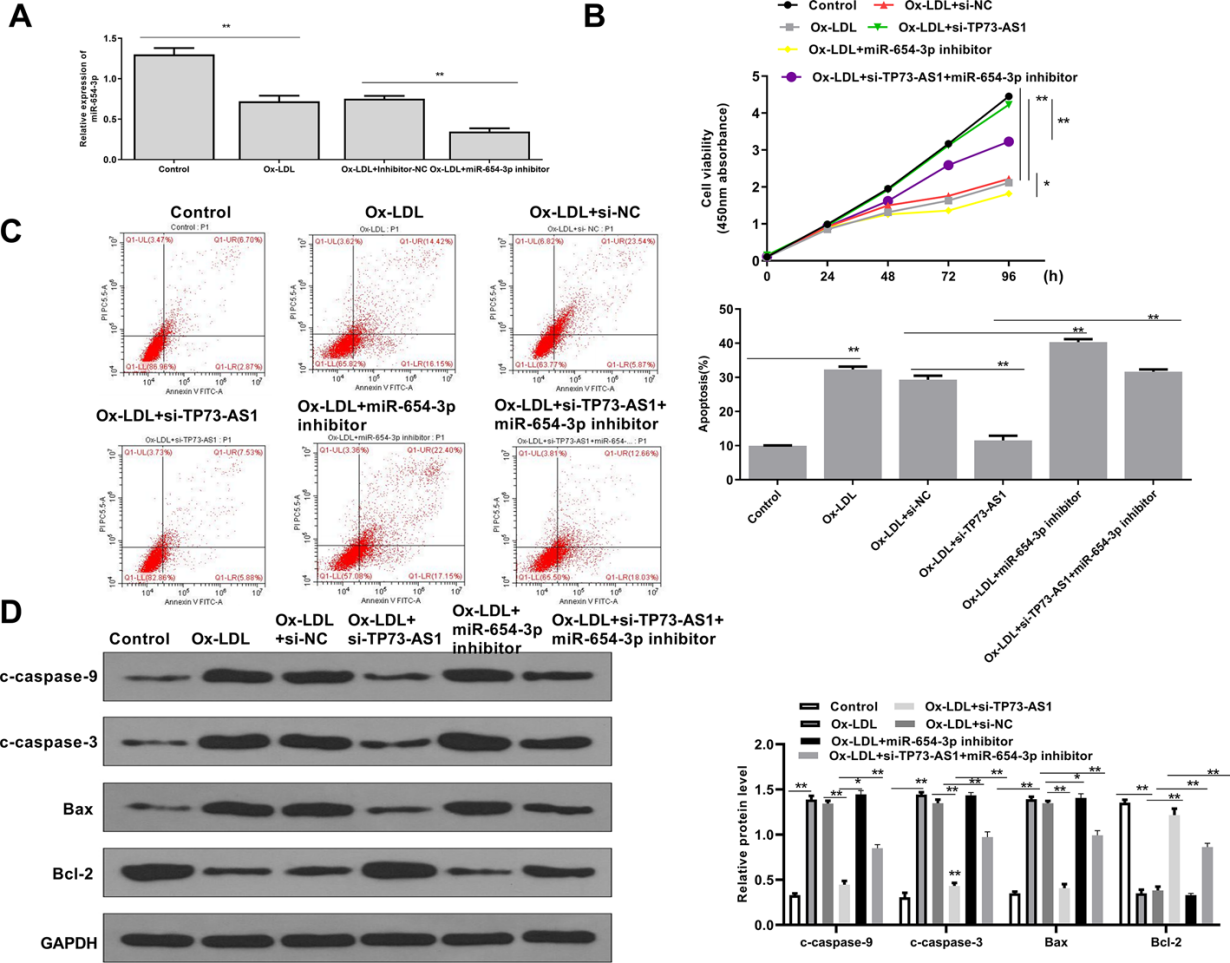
TP73-AS1 knockdown inhibited apoptosis of ox-LDL treated HAECs by targeting miR-654-3p. **A** MiR-654-3p level evaluated by qRT-PCR in HAECs transfected with miR-654-3p inhibitor or inhibitor NC and treated with ox-LDL for 24 h. **B** Cell viability assessed by CCK-8 assay, **C** apoptosis evaluated by flow cytometry and **D** the levels of apoptosis-related proteins detected by Western blot in HEACs with si-TP73-AS1, si-NC, miR-654-3p inhibitor, or co-transfected with si-TP73-AS1 and miR-654-3p inhibitor, and treated with 50 μg/ml ox-LDL for 24 h. *P < 0.05, **P < 0.01

To determine whether the effect of TP73-AS1 in AS was mediated by miR-654-3p, si-TP73-AS1 and miR-654-3p inhibitor were co-transfected into HAECs. Ox-LDL treatment significantly decreased the miR-654-3p level (*p* < 0.01), and miR-654-3p inhibitor further decreased the miR-654-3p level compared with miR-NC in ox-LDL treated HAECs (*p* < 0.01) (Fig. 5A). CCK-8 assay showed that ox-LDL treatment significantly decreased HAECs’ viability (*p* < 0.01), miR-654-3p inhibitor further decreased cell viability of ox-LDL treated HAECs (*p* < 0.05); si-TP73-AS1 promoted viability of ox-LDL treated HAECs (*p* < 0.01), while co-transfection of miR-654-3p inhibitor and si-TP73-AS1 significantly reversed the inhibitory effect of si-TP73-AS1 (*p* < 0.01) (Fig. 5B). Ox-LDL treatment promoted apoptosis of HAECs (*p* < 0.01), and si-TP73-AS1 significantly inhibited apoptosis of ox-LDL treated HAECs (*p* < 0.01). Moreover, miR-654-3p inhibitor further promoted apoptosis of ox-LDL treated HAECs (*p* < 0.01), while co-transfection of miR-654-3p inhibitor and si-TP73-AS1 significantly reversed the inhibitory effect of si-TP73-AS1 on apoptosis of ox-LDL treated HAECs (*p* < 0.01) (Fig. 5C). In addition, miR-654-3p inhibitor further increased the expression of c-caspase-9, c-caspase-3, and Bax and decreased Bcl-3 expression (all *p* < 0.01), while co-transfection of miR-654-3p inhibitor and si-TP73-AS1 significantly reversed the effect of si-TP73-AS1 (all *p* < 0.01) (Fig. 5D). These results revealed that TP73-AS1 knockdown inhibited apoptosis of ox-LDL treated HAECs partially by targeting miR-654-3p.

### AKT3 was identified as a target of miR-654-3p

**Fig. 6:**
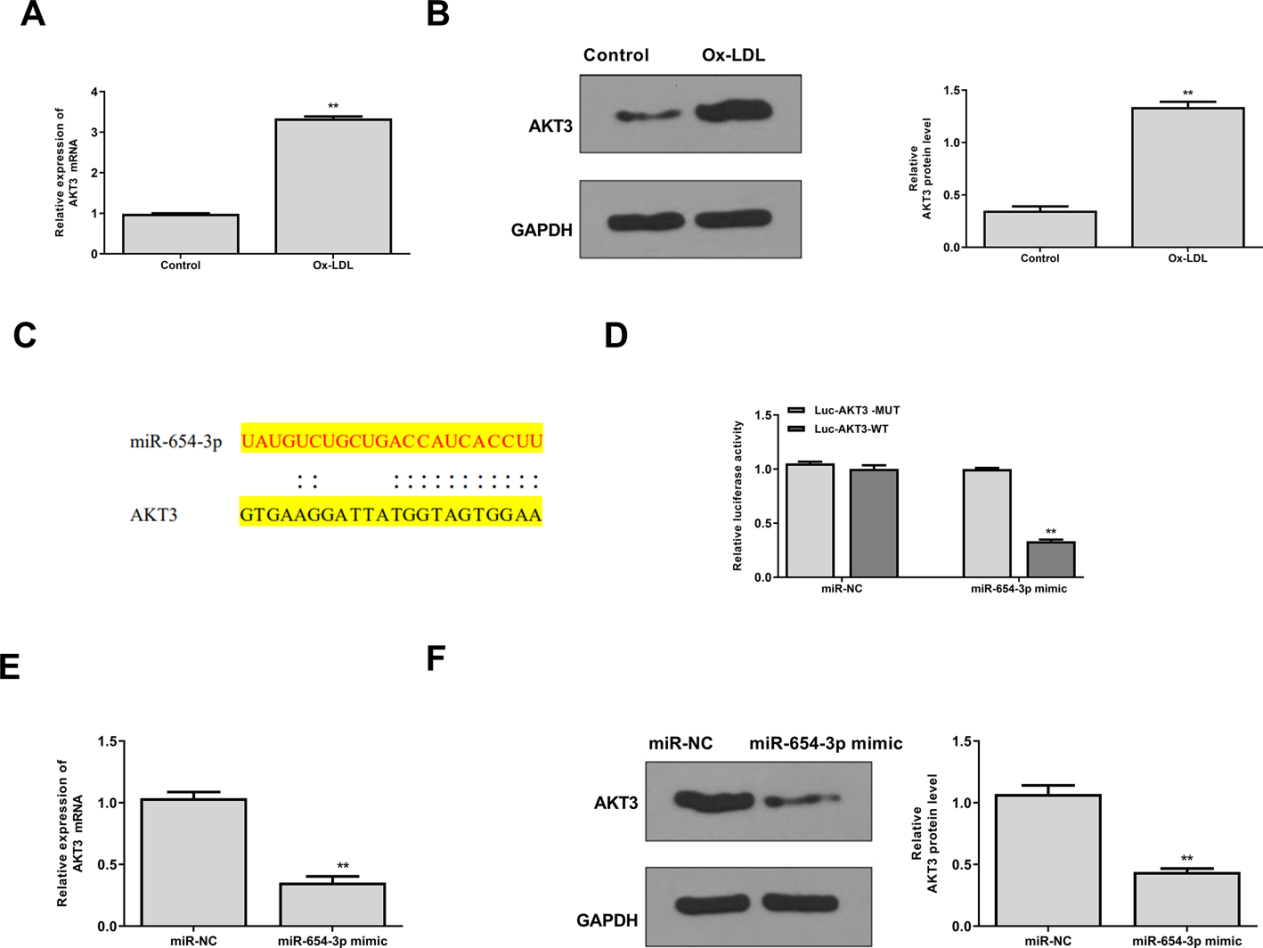
AKT3 was identified as a target of miR-654-3p. **A**, **B** AKT3 level detected by qRT-PCR (**A**) and western blot (**B**) in HAECs were treated with or without 50 μg/ml Ox-LDL for 24 h. **C** The putative binding site between miR-654-3p and AKT3 predicted by StarBase. **D** Results of dual luciferase reporter assay. **E**, **F** AKT level detected by qRT-PCR (**E**) and Western blot (**F**) in HAECs transfected with miR-654-3p mimics or miR-NC. **P < 0.01

Then we explored the downstream mechanism of miR-654-3p and found that ox-LDL treatment significantly increased AKT3 expression compared with the control at both mRNA (*p* < 0.01) (Fig. 6A) and protein levels (*p* < 0.01) (Fig. 6B). The prediction by StarBase revealed a putative binding site between miR-654-3p and AKT3 (Fig. 6C), suggesting that AKT3 might be a target of miR-654-3p. Luciferase reporter assay also confirmed their binding (p < 0.01) (Fig. 6D). In addition, miR-654-3p mimics markedly decreased AKT3 expression (p < 0.01) (Fig. 6E, F) in HAECs. These data suggested that AKT3 was a target of miR-654-3p.

### MiR-654-3p mimics inhibited apoptosis of ox-LDL treated HAECs through targeting AKT3

**Fig. 7:**
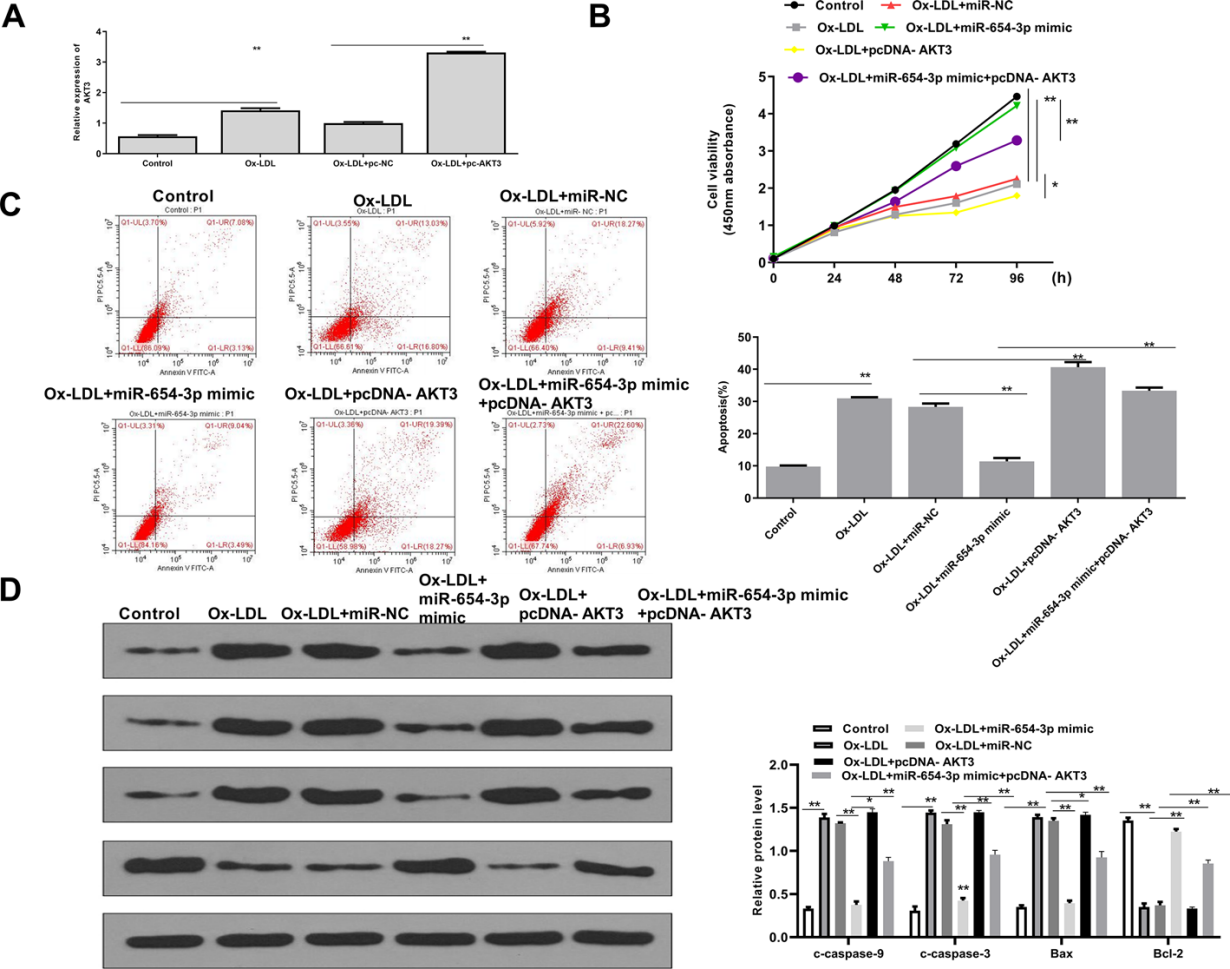
MiR-654-3p mimics inhibited apoptosis of ox-LDL treated HAECs through targeting AKT3. **A** AKT3 level evaluated by qRT-PCR in HAECs transfected with pc-AKT3 or pc-NC, and treated with ox-LDL for 24 h. **B**–**D** (**B**) Cell viability assessed by CCK-8 assay, **C** apoptosis evaluated by flow cytometry and **D** the levels of apoptosis-related proteins detected by Western blot in HAECs transfected with miR-NC, miR-654-3p mimics, pc-AKT3, or co-transfected with miR-654-3p mimics and pc-AKT3, and treated with ox-LDL for 24 h. * P < 0.05, ** P < 0.01

Next, we explored whether the inhibitory effect of miR-654-3p mimics on ox-LDL induced HAEC apoptosis was mediated by AKT3. QRT-PCR analysis showed that ox-LDL treatment increased the AKT3 level compared with the control group (*p* < 0.01), and pc-AKT3 (overexpression of AKT3) significantly further increased the AKT3 level compared with pc-NC (*p* < 0.01) (Fig. 7A). CCK-8 assay showed that overexpression of AKT3 further decreased the viability of ox-LDL treated HAECs (*p* < 0.05), and co-transfection significantly reversed the inhibitory effect of miR-654-3p mimics (*p* < 0.01) (Fig. 7B). Meanwhile, AKT3 overexpression further promoted apoptosis of ox-LDL treated HAECs compared with pc-NC (*p* < 0.01), while co-transfection significantly reversed the inhibitory effect of miR-654-3p mimics (*p* < 0.01) (Fig. 7C). In addition, AKT3 overexpression significantly increased the protein levels of c-caspase-9 (*p* < 0.05), c-caspase-3 (*p* < 0.01), and Bax (*p* < 0.05), while it decreased Bcl-2 expression (*p* < 0.01) compared with miR-654-3p mimics in ox-LDL treated HAECs, while co-transfection of miR-654-3p mimics and pc-AKT3 significantly reversed the effect of miR-654-3p mimics (*p* < 0.01) (Fig. 7D). These results suggested that miR-654-3p mimics inhibited apoptosis of ox-LDL treated HAECs through targeting AKT3.

## Discussion

In the last decades, the chronic inflammatory disease AS has become a global clinical problem [26]. Therefore, there is an urgent need to explore the specific pathogenic mechanisms in AS progression to help develop effective therapeutic targets and relieve patients’ pain. Here, we found that TP73-AS1 was upregulated in ox-LDL treated HAECs. Our results revealed a complete mechanism of TP73-AS1 in AS development. Specially, TP73-AS1 promoted apoptosis of ox-LDL treated HAECs by upregulating AKT3 expression via directly sponging miR-654-3p.

Recently, a number of lncRNAs have been identified to be closely involved in cell proliferation, apoptosis, and the immune response during AS occurrence and might be considered as potential diagnostic and therapeutic biomarkers. For example, FA2H-2 knockdown activated inflammation and inhibited autophagy flux, leading to aggravation of ox-LDL induced inflammatory responses in human aorta vascular smooth muscle cells [27]. MALAT1 was robustly expressed in the macrophages of rats with diabetic atherosclerosis, and low-dose sinapic acid abated the pyroptosis of macrophages through inhibiting MALAT1 expression [28]. TUG1 was upregulated during AS, and its knockdown significantly inhibited the inflammatory response and attenuated atherosclerotic lesions in a mouse model [29]. MALAT1 is downregulated in ox-LDL treated vascular endothelial cells, and its downregulation promoted dendritic cell maturation in AS development [30]. lncRNA-ATB promoted viability, migration and angiogenesis of human microvascular endothelial cells, and might be considered as a potential biomarker for AS [31]. Although many lncRNAs have been revealed to be associated with AS development, more effective and specific molecular targets are still necessary. In this study, we found that TP73-AS1 downregulation effectively inhibited AS progression in vitro, suggesting that TP73-AS1 might be another potential target for AS treatment.

LncRNAs always function as sponges of miRNAs to regulate biological processes in the development of human cardiovascular diseases [32, 33]. Silencing H19 attenuated the inflammatory response by sponging miR-130b in ox-LDL-treated Raw264.7 cells [34]. LEF1-AS1 modulated the proliferation of vascular smooth muscle cells through targeting miR-544a [35]. MALAT1 suppression protected endothelium against ox-LDL induced inflammation by inhibiting miR-181b expression [36]. LncRNA ZFAS1 enhanced inflammatory responses in AS by directly sponging miR-654-3p to upregulate ADAM10 and RAB22A [15]. Hence, we hypothesized that TP73-AS1 might directly sponge miR-654-3p to affect AS progression. Both luciferase reporter and RIP assays confirmed their relationship. Rescue experiments demonstrated that co-transfection of miR-654-3p inhibitor and si-TP73-AS1 attenuated si-TP73-AS1 induced inhibition on cell apoptosis. These data indicated that the effect of TP73-AS1 in AS was partially mediated by miR-654-3p.

Previous studies demonstrated that AKT3 could promote tumor development in different human cancers [37]. Importantly, AKT3 was also highly expressed in AS, and loss of AKT3 led to inhibition of AS development [38]. Consistent with these reports, our results confirmed that AKT3 was significantly upregulated in ox-LDL treated HAECs. Interestingly, a previous study reported AKT3 as a target of miR-654-3p, and the miR-654-3p/AKT3 axis was closely involved in the proliferation and invasion of ovarian cancer cells [39]. In the present study, rescue experiments confirmed the role of the miR-654-3p/AKT3 axis in AS.

High-fat diet-induced animal models have been a widely used strategy to determine the function of lncRNAs/miRNAs in AS development [22, 40]. The impact of TP73-AS1 in AS progression should be confirmed using these models in the future.

## Conclusion

In summary, we investigated the role of TP73-AS1 in the apoptosis of HAECs and revealed that TP73-AS1 downregulation effectively inhibited apoptosis of ox-LDL treated HAECs through regulating the miR-654-3p/AKT3 axis. Our study suggested that TP73-AS1 might be a potential target for AS treatment.

## Data Availability

The datasets analyzed during the current study are available from the corresponding author on reasonable request.

## Abbreviations

AS: Atherosclerosis
ox-LDL: Oxidized low-density lipoprotein
CVDs: Cardiovascular diseases
lncRNAs: Long non-coding RNAs
miRNAs: MicroRNAs
